# Complete Mitochondrial Genome of *Porites cylindrica* From the Xisha Islands: Characterization and Comparative Mitogenomics of the Genus

**DOI:** 10.1002/ece3.73297

**Published:** 2026-03-18

**Authors:** Shuwen Jia, Tongtong Shen, Wenqi Cai, Jian Zhang, Yi Wang, Zefu Cai, Jie Shen, Shiquan Chen

**Affiliations:** ^1^ Qukou Scientific Research Base, Institute of Marine Ecology Hainan Academy of Ocean and Fisheries Sciences Haikou China; ^2^ Key Laboratory for Coastal Marine Eco‐Environment Process and Carbon Sink of Hainan Province, Yazhou Bay Innovation Institute College of Ecology and Environment, Hainan Tropical Ocean University Sanya China; ^3^ Key Laboratory of Utilization and Conservation for Tropical Marine Bioresources (Hainan Tropical Ocean University) Ministry of Education Sanya China

**Keywords:** coral phylogeny, mitochondrial DNA, mitochondrial genome evolution, *Porites cylindrica*

## Abstract

Characterizing the mitochondrial genomes of reef‐building corals, which are crucial for marine ecosystems, offers profound insights into their phylogenetic relationships and evolutionary history. The genus *Porites* is extensively distributed across the Indo‐Pacific region. Nevertheless, few analyses of the mitochondrial genome characteristics of *Porites* have been performed. Here, we report the sequencing and de novo assembly of the complete mitochondrial genome of *Porites cylindrica* , a dominant Indo‐Pacific reef‐building coral. Subsequently, a comparative phylogenetic analysis was performed using this new assembly and 12 publicly available mitogenomes derived from 10 *Porites* species. Mitochondrial genome size was highly conserved, ranging from 18,628 to 18,658 bp across the dataset, and contained 13 protein‐coding genes, two rRNA genes, and 1–12 different numbers of tRNAs. Sixteen mutation hotspot regions were identified (ND5‐exon1, trnE‐ND1, ND1, ND1‐CYTB, CYTB, CYTB‐trnM, trnM, ATP6, ATP6‐ND4, ND4, ND4‐rrnS, rrnS, rrnS‐COX3, COX3, COX2 and ND5‐exon2), and they can serve as effective molecular markers for further phylogenetic analysis and species identification. In addition, microsatellite loci in mitochondrial genomes were highly conserved among various species. Strong purifying selection was observed in the 13 protein‐coding genes of *Porites*. The phylogenetic results support the monophyly of *Porites* among scleractinian corals, and this genus has diverged into two clades. This study reports the initial assembly and annotation of *P. cylindrica* , thus providing valuable data for future research on the mitochondrial genomes of *Porites*.

## Graphical abstract

In this study, we sequenced the dominant species, *Porites andrewsi* , in the Xisha Islands of the South China Sea, assembled its mitochondrial genome, and analyzed the evolution of the mitochondrial genome of Porites species in combination with 12 other mitochondrial genomes from nine other species. Our results lay the foundation for further research on the mitochondrial genome evolution process and patterns of Porites and other members of the Poritidae family.

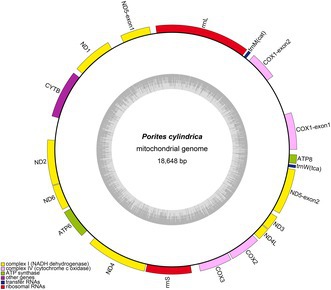

## 1 Introduction

Mitochondria play a crucial role in the process of cell metabolism and energy conversion and are key organelles involved in biological responses to environmental changes (Baris et al. 2016; Chan 2020; Shang et al. 2022). In addition to the genetic material in the nuclear genome, mitochondria possess their own independent genetic material, the mitochondrial genome. Probing the genetic changes in the mitochondrial genome not only advances our understanding of species evolution and phylogeny (Bernt, Braband, et al. 2013; Bernt, Donath, et al. 2013) but also provides valuable insights for exploring biological adaptation (Luz et al. 2017; Tsilingiris et al. 2021). However, despite the importance of mitochondrial genome diversity, evolution, and adaptation, our knowledge of these factors in scleractinian corals remains limited.

The genus *Porites* belongs to the family Poritidae and is among the most diverse genera of reef‐building corals. To date, 68 valid species of *Porites* have been accepted by the taxonomic community, as recorded in the World Register of Marine Species, and they are widely distributed across the Indo‐Pacific (World Register of Marine Species, https://www.marinespecies.org/aphia.php?p=taxlist, Accessed 2025‐10‐10). Although numerous mitochondrial genome sequences of *Porites* have been reported (Celis et al. 2016; Niu, Lin, et al. 2016; Terraneo et al. 2018), studies focusing on their mitochondrial genome structure and characteristics remain limited (Paz‐García et al. 2016). *Porites cylindrica* Dana 1846 is widely distributed in tropical and subtropical regions of the Indo‐Pacific Ocean, where it acts as a dominant reef‐building coral species (Combosch et al. 2024; Kitano et al. 2014). While a mitochondrial genome sequence is available for *P. cylindrica* (Fiji; accession: OQ037789), its annotation as a linear molecule contradicts the standard circular model for scleractinian corals, suggesting that the data should be interpreted with caution until verified. The record also lacks structural annotation in NCBI. Overall, comparative mitogenomic analyses are lacking for this species and for the genus *Porites*. Therefore, to resolve the uncertainty regarding its mitochondrial architecture and to facilitate comprehensive genus‐level comparisons, we sequenced, assembled, and characterized the mitochondrial genome of *P. cylindrica* from the Xisha Islands. By integrating this new mitochondrial genome with all available *Porites* mitogenomes from public databases, we performed a detailed comparative analysis of 13 mitogenomes spanning 10 species. This study provides the first circular mitogenome for *P. cylindrica* and offers new insights into the structural conservation and evolutionary relationships within the genus *Porites*.

## 2 Materials and Methods

### 2.1 Sample Collection and DNA Extraction

On August 12, 2023, five *P. cylindrica* samples (5‐cm long) were manually collected from Xishazhou (112.2384687 E, 16.97453230 N) of the Xisha Islands by scuba divers using chisels and hammers and identified according to the description of *P. cylindrica* in Corals of the World (http://www.coralsoftheworld.org/). The collected samples were washed with filtered seawater, rapidly frozen in liquid nitrogen, and stored at −80°C freezer. Total DNA was extracted from the coral tissues using a Universal Genomic DNA Kit (Kangwei Century Biotechnology Co. Ltd., Beijing, China); the integrity of the coral DNA was checked using an Agilent Fragment Analyzer 5400 (Agilent Technologies, Santa Clara, CA, USA).

### 2.2 High‐Throughput Sequencing, Mitochondrial Genome Assembly, and Mitochondrial Genome Annotation

Total DNA of *P. cylindrica* was sent to Huitong Biotechnology (Shenzhen, China) for library sequencing. DNA sequencing library construction was performed using a Nextera XT DNA library preparation kit (Illumina, San Diego, CA, USA), and then sequencing was performed using the MGIDNBSEQ‐T7 platform. A total of 25.67G raw base and 171,110,472 raw reads were obtained (GenBank accession number PRJNA1191632). The data were filtered using fastp v 0.23.4 (https://github.com/OpenGene/fastp; Chen et al. 2018). Subsequently, de novo assembly of the mitochondrial genome was conducted using GetOrganelle v1.7.7.0 (Jin et al. 2020) with *k*‐mer settings of 21, 45, 65, 85, and 105. The mitochondrial contigs within the assembled sequences were identified by aligning with the reference genome of *Porites lobata* Dana, 1846 (NC_030186). The assembly graph was processed with Bandage v 0.8.1 (Wick et al. 2015) to remove redundant contigs. The remaining contig was edited into a circular molecule, which was determined to be the complete mitochondrial genome. To assess assembly quality and coverage, clean reads were mapped back to the assembled mitochondrial sequence using BWA v 0.7.17‐r1188 (Li 2013), and sequencing depth was calculated using samtools v 1.9 (Li et al. 2009). A coverage plot was generated using a Python script, with the mitochondrial genome position as the *x*‐axis and sequencing depth as the *y*‐axis (Figure S1). The complete mitochondrial genome obtained through assembly was annotated using MITOS2 (https://usegalaxy.eu/?tool_id=toolshed.g2.bx.psu.edu%2Frepos%2Fiuc%2Fmitos2%2Fmitos2%2F2.1.9%2Bgalaxy0&version=latest), and the annotation results were manually refined. Finally, Organellar Genome DRAW v1.2 (Greiner et al. 2019) was utilized to generate the mitochondrial genome circular diagram online. The base composition and amino acid content of mitochondrial genomes were analyzed using MEGA v 6.0 (Tamura et al. 2013). The mitochondrial genome sequence obtained in this study has been uploaded to the NCBI database under the accession number PQ666634.

### 2.3 Analysis of Mitochondrial Genome Characteristics

Twelve mitochondrial sequences from 10 *Porites* species ( *P. cylindrica* OZ037789; *P. lobata* NC_030186.1; *P. lobata* KU761954.1; *Porites okinawensis* Veron, 1990 NC_015644.1; *Porites harrisoni* Veron, 2000 NC_037435.1; *Porites lutea* Milne Edwards & Haime, 1851 NC_029695.1; *Porites rus* Forskål, 1775 LN864762.1; *Porites fontanesii* Benzoni & Stefani, 2012 NC_037434.1; *Porites porites* Pallas, 1766 NC_008166.1; *Porites panamensis* Verrill, 1866 KU761953; *P. panamensis* NC_024182.1; and *Porites sverdrupi* Durham, 1947 KU956960.1) were downloaded from GenBank. The unannotated sequence of *P. cylindrica* OZ037789 was annotated using the same methods applied to *P. cylindrica* PQ666634 (Figure S2). Using the mitochondrial genome of *P. cylindrica* PQ666634 as a reference, the sequences of the 13 mitochondrial genomes were compared using Blast v 2.10.1 (Cock et al. 2015).

The relative synonymous codon usage (RSCU) and number of mitochondrial genes were calculated using CodonW v1.3 (Sharp et al. 1986). Microsatellite sequences, or single‐sequence repeats (SSRs), were identified using MISA (https://webblast.ipk‐gatersleben.de/misa/), with minimum repeat counts of 10, 5, 4, 3, 3, and 3 for mononucleotide, dinucleotide, trinucleotide, tetranucleotide, pentanucleotide, and hexanucleotide sequences, respectively (Beier et al. 2017). Long repeats were identified in REPuter (https://bibiserv.cebitec.uni‐bielefeld.de/reputer/; Kurtz et al. 2001), with the minimum repeat size set to 15 bp.

The localization and characterization of the putative mitochondrial control region (CR) were attempted through two complementary strategies. First, an ab initio prediction was performed using the MITOS2 (Donath et al. 2019) with parameters set to ‐c 4 ‐r refseq89m. Second, based on the common structural criterion in Crustacean mitogenomics where the CR often constitutes the largest non‐coding interval (Baeza 2022), we identified the longest intergenic spacer within the assembly. This region, located between tRNA‐Glu and ND5 (positions 8520–9088, 569 bp), was designated as the structural candidate CR. This candidate CR was then subjected to detailed characterization. The candidate CR was then scanned for microsatellites (SSRs) using the MISA (Beier et al. 2017) and parameters as previously described. Tandem repeat detection was performed using Tandem Repeats Finder v4.09 (Benson 1999) with parameters set as: 2 7 7 80 10 50 2000 ‐f ‐d ‐m. The potential for forming stable secondary structures was assessed using the RNA fold web server under default settings.

### 2.4 Analysis of Mitochondrial Genome Selection Pressure

Using *P. cylindrica* PQ666634 as a reference, the mitochondrial genomes of 10 *Porites* species were aligned in MAFFT v7.429 (Katoh and Standley 2016) using the FFT‐NS‐2 strategy. The nucleotide variability (Pi) of the entire mitochondrial genome was analyzed using DnaSp v6 (Rozas et al. 2017) based on a sliding window length of 300 bp and a step size of 25 bp. Sequences from 13 protein‐coding genes (PCGs) were initially processed using Blastn v2.9.0+ with parameters set to ‐evalue 1e‐5 and ‐max_target_seqs 1 to align the target species' protein sequences to the reference, with the best‐matched homologous gene pairs retained. The protein and nucleotide sequences of both the target and reference species were then combined and formatted using ParaAT v2.0 (Zhang et al. 2012). Nonsynonymous (Ka) and synonymous (Ks) substitution rates were estimated using KaKs_Calculator v2.0 (Wang et al. 2010) with the Yang and Nielsen (2000) model (YN method). This model accounts for transition/transversion rate bias and codon usage bias, providing robust estimates for closely related sequences. Finally, R v 4.2.0 was used to construct a Ka/Ks heatmap.

### 2.5 Genomic Collinearity and Phylogenetic Analysis

To determine the phylogenetic position of *P. cylindrica* , a mitochondrial genome‐based phylogenetic analysis was conducted. Following the taxonomic framework of recent mitogenomic studies (Tian et al. 2022; Jia et al. 2025), *Corallimorphus profundus* and *Corynactis californica* were designated as outgroups. The nucleotide sequences of 13 protein‐coding genes (*ATP6*, *ATP8*, *COX1*, *COX2*, *COX3*, *CYTB*, *ND1*, *ND2*, *ND3*, *ND4*, *ND4L*, *ND5*, and *ND6*) were extracted from the 60 samples (58 scleractinian corals and 2 outgroups). Each of the 13 PCGs was aligned independently using MAFFT v 7.429 (Katoh and Standley 2016) with the FFT‐NS‐2 strategy. To obtain a robust alignment for phylogenetic inference, each gene alignment was processed using Gblocks v 0.91b (Castresana 2000). The program was run with the ‐t=DNA option specified, while all other parameters were kept at their default settings to remove ambiguously aligned regions, primarily those containing gaps. The resulting high‐confidence alignments for all genes were then concatenated into a single supermatrix. Phylogenetic trees were reconstructed using both Maximum Likelihood (ML) and Bayesian Inference (BI) methods. ML analysis was performed using IQ‐TREE v 1.6.12 (Nguyen et al. 2015). The best‐fit nucleotide substitution model was determined by the built‐in ModelFinder program based on the Bayesian Information Criterion (BIC). The selected model for the entire concatenated dataset was TVM+F+I+G4. Branch support was assessed with 1000 standard non‐parametric bootstrap replicates. BI analysis was conducted using MrBayes v 3.2.6 within the PhyloSuite v1.2.1 platform (Zhang et al. 2020). The best‐fit model for the BI analysis, selected independently by PhyloSuite's ModelFinder under BIC, was GTR+F+I+G4. A Markov Chain Monte Carlo (MCMC) analysis was run with four chains (two cold and two heated) for 600,000 generations, sampling trees and parameters every 1000 generations. The first 25% of sampled trees were discarded as burn‐in, and a majority‐rule consensus tree was constructed from the remaining trees to estimate Bayesian Posterior Probabilities. The topologies from the ML and BI analyses were combined, and the final phylogenetic tree was visualized and annotated using Adobe Illustrator CS6.

The rRNA gene positions of the 10 *Porites* species were obtained using Python scripts. The best base substitution model was determined using IQ‐TREE's built‐in ModelFinder, and it was found to be K3Pu+F+I according to the BIC optimal nucleic acid replacement model. Bootstrapping was set to Ultrafast, and the number of bootstraps was set to 1000. Finally, a collinearity analysis chart was constructed using AliTV (https://alitvteam.github.io/AliTV/d3/AliTV.html) (Ankenbrand et al. 2017).

The mitochondrial arrangement order for each of the 13 mitochondrial genomes was determined using Python scripts, and a gene rearrangement map was prepared in Adobe Illustrator CS6 (Adobe, USA).

## 3 Results

### 3.1 Characteristics of Mitochondrial Genomes in *Porites* Species

**FIGURE 1:**
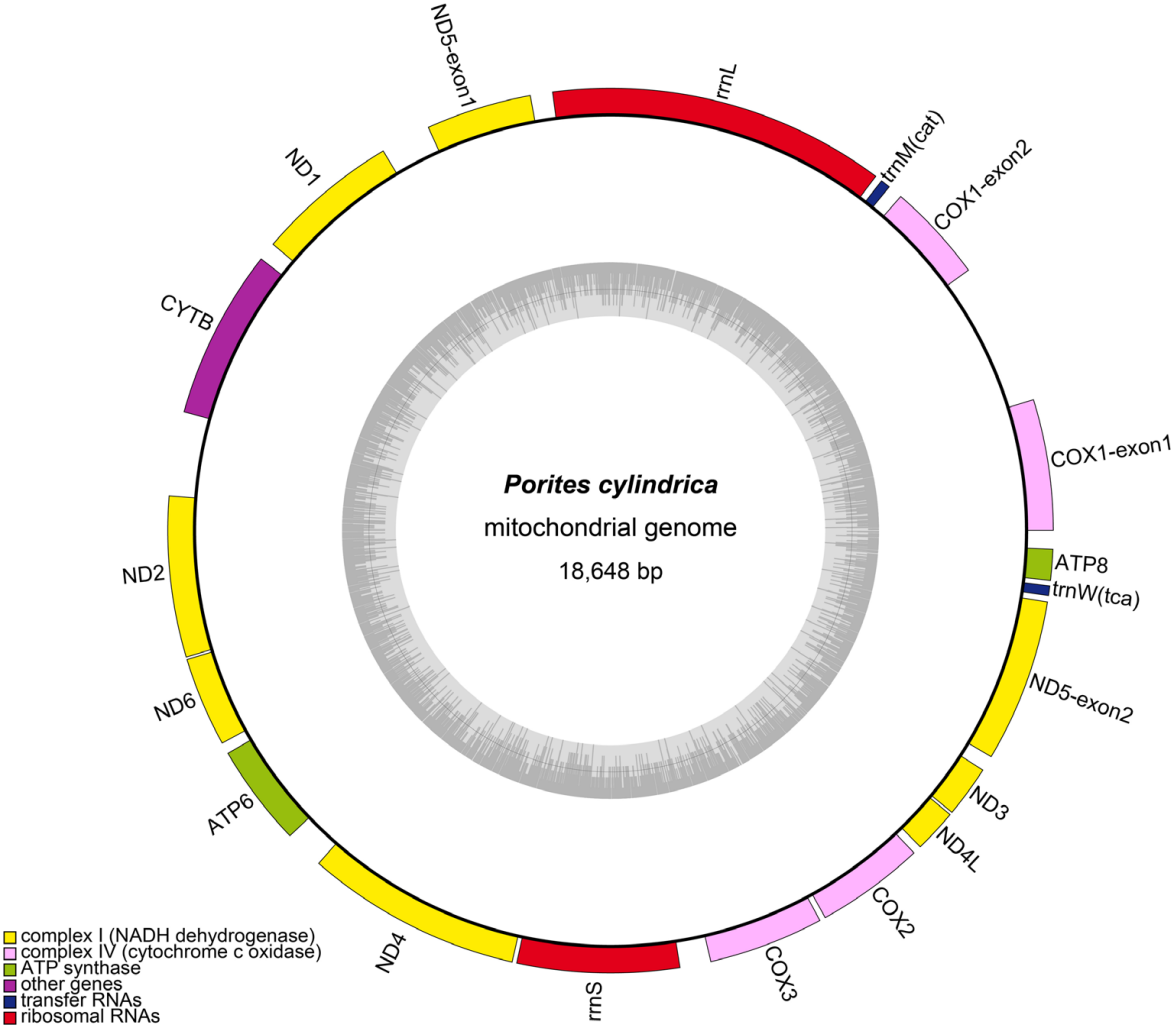
Circular map of the complete mitochondrial genome of *Porites cylindrica* . Genes with different functions are distinguished by different colors (NADH dehydrogenase: Yellow; cytochrome c oxidase: Purple; ATP synthase: Green; tRNA genes: Blue; rRNA genes: Red). The light gray area on the inner circle represents the GC content, and the dark gray area represents the AT content.

The complete mitochondrial genome of *P. cylindrica* (accession number: PQ666634) was successfully assembled as a single circular molecule using GetOrganelle v1.7.7.0 (Jin et al. 2020). The mitochondrial genome had a total length of 18,648 bp, GC content of 36.25%, and base composition of A (25.97%), T (37.78%), C (13.23%), and G (23.02%) (Figure 1; Table 1). All genes were encoded on the heavy strand, including 13 protein‐coding genes, two tRNA genes, and two rRNA genes. Coverage analysis confirmed the high quality and completeness of the assembly, showing a uniform sequencing depth with an average of 843.68× and a minimum of 281× (Figure S1).

**TABLE 1:** Composition of 13 mitochondrial genomes from 10 *Porites* species.

Species	Size/bp	G+C content/%	Number of genes	Number of PCGs	Number of tRNA	Number of rRNA
*Porites cylindrica* PQ666634	18,648	36.25	17	13	2	2
*Porites cylindrica* OZ037789	18,647	36.22	18	13	3	2
*Porites harrisoni* NC_037435.1	18,630	36.34	17	13	2	2
*Porites fontanesii* NC_037434.1	18,658	36.64	17	13	2	2
*Porites lutea* NC_029695.1	18,646	36.26	23	13	8	2
*Porites porites* NC_008166.1	18,648	36.27	17	13	2	2
*Porites lobata* NC_030186.1	18,647	36.24	17	13	2	2
*Porites lobata* KU761954.1	18,647	36.20	16	13	1	2
*Porites panamensis* NC_024182.1	18,628	36.28	16	13	1	2
*Porites panamensis* KU761953	18,644	36.27	16	13	1	2
*Porites sverdrupi* KU956960.1	18,628	36.29	17	13	2	2
*Porites okinawensis* NC_015644.1	18,647	36.20	16	13	1	2
*Porites rus* LN864762.1	18,647	36.23	27	13	12	2

The 13 mitochondrial genomes of the *Porites* species ranged from 18,628 to 18,658 bp, with *P. panamensis* and *P. sverdrupi* having the smallest mitochondrial genome (18,628 bp) and *P. fontanesii* having the largest mitochondrial genome (18,658 bp). The GC content of the genomes was between 36.2% and 36.64%, with *P. lobata* and *P. okinawensis* mitochondria presenting the lowest GC content and *P. fontanesii* presenting the highest. The number of genes in the *Porites* species ranged from 16 to 27, with 13 PCGs, 2 rRNA, and 1–12 tRNA genes (Table 1).

### 3.2 Repetitive Sequences in the Mitochondrial Genomes of *Porites* Species

**FIGURE 2:**
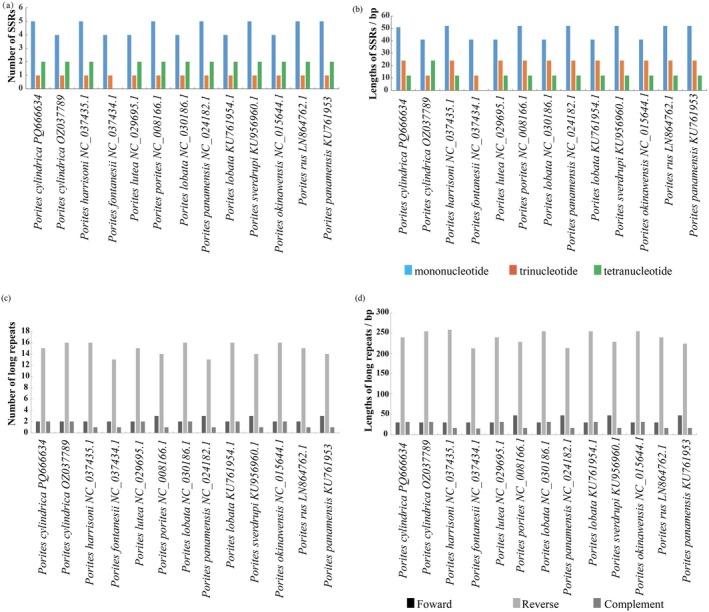
Repeat sequence analysis of mitochondrial genomes from 10 *Porites* species. (a) Distribution of different microsatellite loci. (b) Length distribution of long repeats at microsatellite loci. (c) Distribution of different types of long repeats. (d) Length distribution of long repeats.

In this study, 96 SSR loci were detected in the 13 mitochondrial genomes of the *Porites* species (Figure 2; Table S1). The total number of SSRs between different species did not change significantly, except for that in *P. fontanesii*, which exhibited only five SSR loci, whereas the SSR number variation for other species ranged from seven to eight. Most of these mitochondrial SSRs were mononucleotides, accounting for 61.46% of all SSRs, followed by tetranucleotides (25.00%) and trinucleotides (13.54%). No dinucleotide, pentanucleotide, or hexanucleotide repeat motifs were detected. The A/T mononucleotide repeat motifs were the most abundant SSR loci, accounting for 94.92% of the mononucleotides. Only three G/C repeat mononucleotide motifs were detected, which accounted for 5.08% of the mononucleotides present. Most SSRs (79.17%) were distributed in PCGs, whereas only 20.83% were located in intergenic spacers. Two hundred‐and forty‐two long repeat sequences were detected in the 13 mitochondrial genomes, including 30 forward, 193 reverse, and 19 complementary repeats (Figure 2; Table S2).

The ab initio annotation by MITOS2 predicted a putative CR; however, this region overlapped the coding sequence of the conserved *cox2* gene, an arrangement deemed biologically implausible for an essential respiratory gene. Therefore, the longest intergenic spacer (569 bp between tRNA‐Glu and ND5) was analyzed as the structural candidate CR. Detailed analysis revealed a remarkably simple architecture: only a single mononucleotide microsatellite (T)_10_ was identified using MISA, and no tandem repeats were detected by Tandem Repeats Finder. Furthermore, secondary structure prediction via RNAfold did not support the formation of a stable, complex hairpin structure typical of canonical metazoan mitochondrial control regions.

### 3.3 Usage Preference of *Porites* Mitochondrial Genome Codons

**FIGURE 3:**
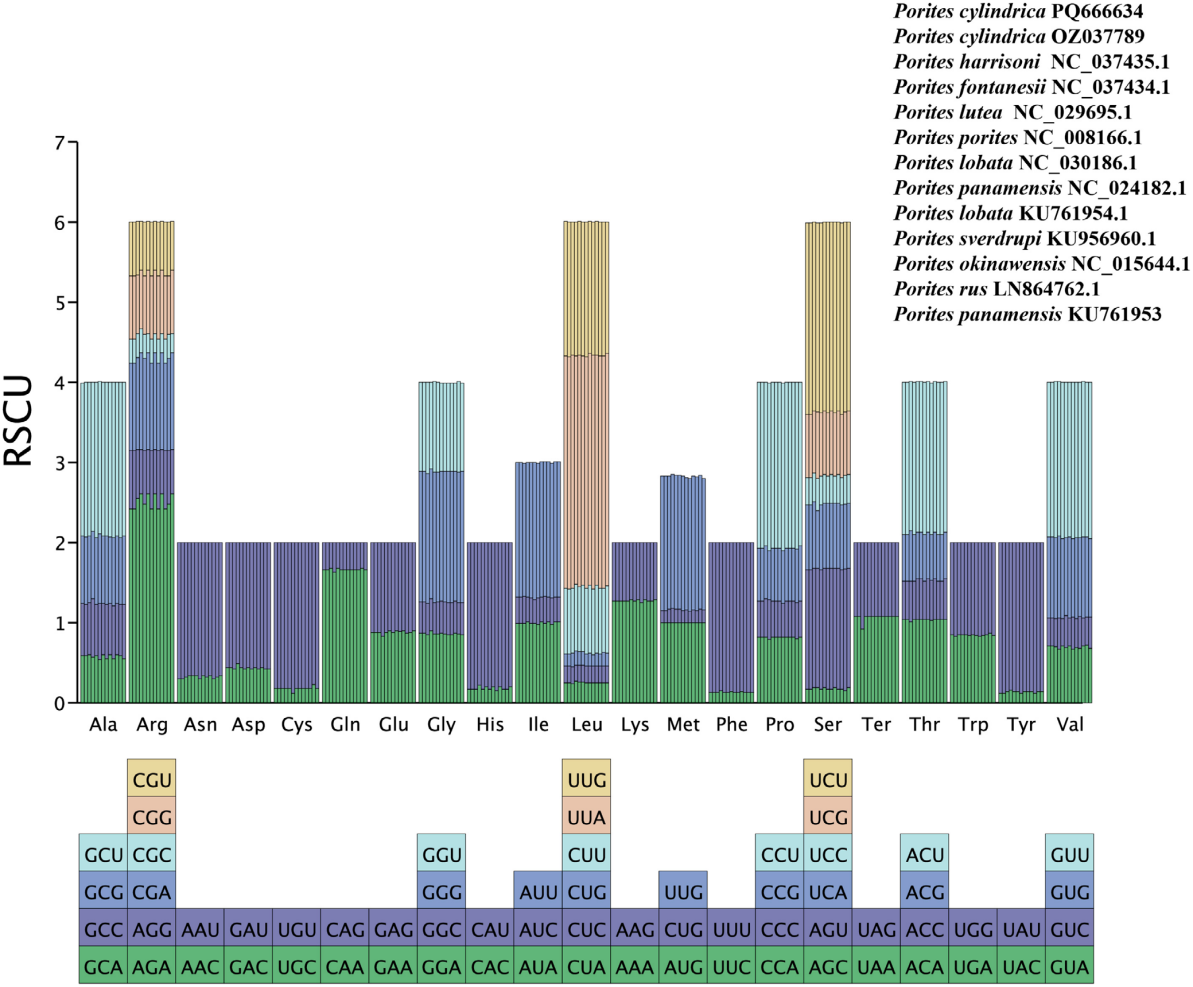
Amino acid synonymous codon usage based on 13 protein‐coding genes of 13 complete mitochondrial genomes of 10 *Porites* species. The synonymous codons of each amino acid are presented as bar charts in different colors, and the color labels are located below the bar charts. Each bar represents a mitochondrial genome, and from left to right, they are *P. cylindrica* PQ666634, *P. cylindrica* OZ037789, *P. harrisoni* NC 037435.1, *P. fontanesii* NC 037434.1, *P. lutea* NC 029695.1, *P. porites* NC 008166.1, *P. lobata* NC 030186.1, *P. panamensis* NC 024182.1, *P. lobata* KU761954.1, *P. sverdrupi* KU956960.1, *P. okinawensis* NC 015644.1, *P. rus* LN864762.1, *P. panamensis* KU761953.

Analyses of mitochondrial protein‐coding genes across 10 *Porites* species revealed a conserved codon usage pattern. As shown in Figure 3 and Table S3, codon usage preferences were highly conserved across all species. Consistent with the high AT content of the mitochondrial genome, the majority of preferred codons (RSCU > 1) terminated in A or T. The most pronounced bias was observed for leucine, which predominantly utilized the UUA codon (RSCU range: 2.85–2.92). Notable codon biases were also evident for arginine (AGA, RSCU: 2.42–2.61) and serine (UCU, RSCU: 2.36–2.39).

### 3.4 Comparative Mitochondrial Genomic Analysis

**FIGURE 4:**
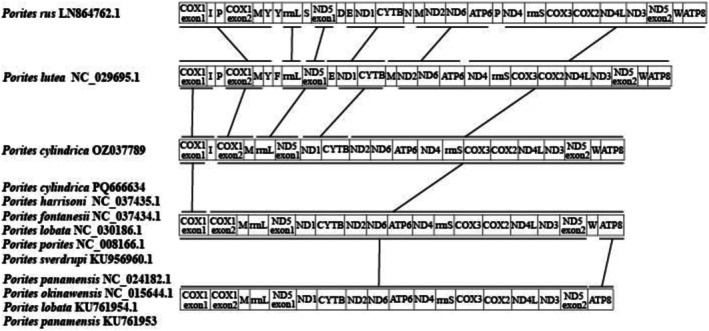
Gene rearrangement analysis of 13 complete mitochondrial genomes of 10 *Porites* species.

**FIGURE 5:**
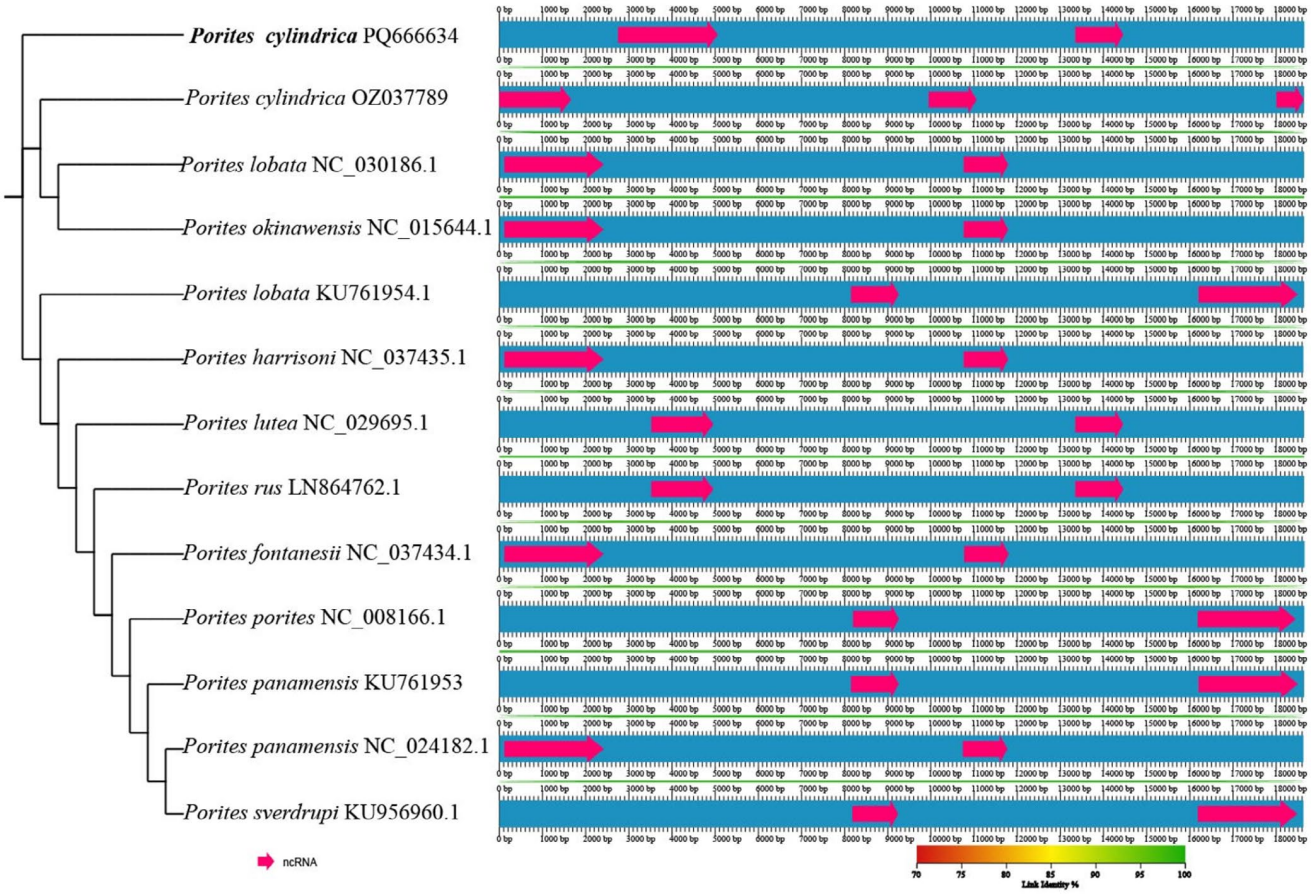
Collinearity analysis of 13 complete mitochondrial genomes of 10 *Porites* species.

Analysis of the mitochondrial genomes of the 10 *Porites* species showed that no gene rearrangement occurred within the genus (Figure 4). However, sequence differences mainly occurred in the type and number of tRNA. Four type and number patterns of tRNA were identified in the genus *Porites*. The patterns in *P. harrisoni* , *P. fontanesii*, *P. lobata* (NC_030186.1), *P. porites* , and *P. sverdrupi* were consistent and the most common among the 13 mitochondrial genomes. The tRNA pattern in *P. cylindrica* and *P. lutea* was also consistent. Notably, mitochondrial genes across different samples of the same species can exhibit inconsistent patterns; for example, the patterns differed between *P. lobata* NC030186.1 and *P. lobata* KU761954.1. The results of the collinearity analysis of the 13 mitochondrial genomes and allied species, with *P. cylindrica* PQ666634 as the reference species, are shown in Figure 5. These results were similar to those of the gene rearrangements, with both showing that sequences of the same species were not necessarily clustered together.

### 3.5 Phylogenetic Analysis of *Porites*

**FIGURE 6:**
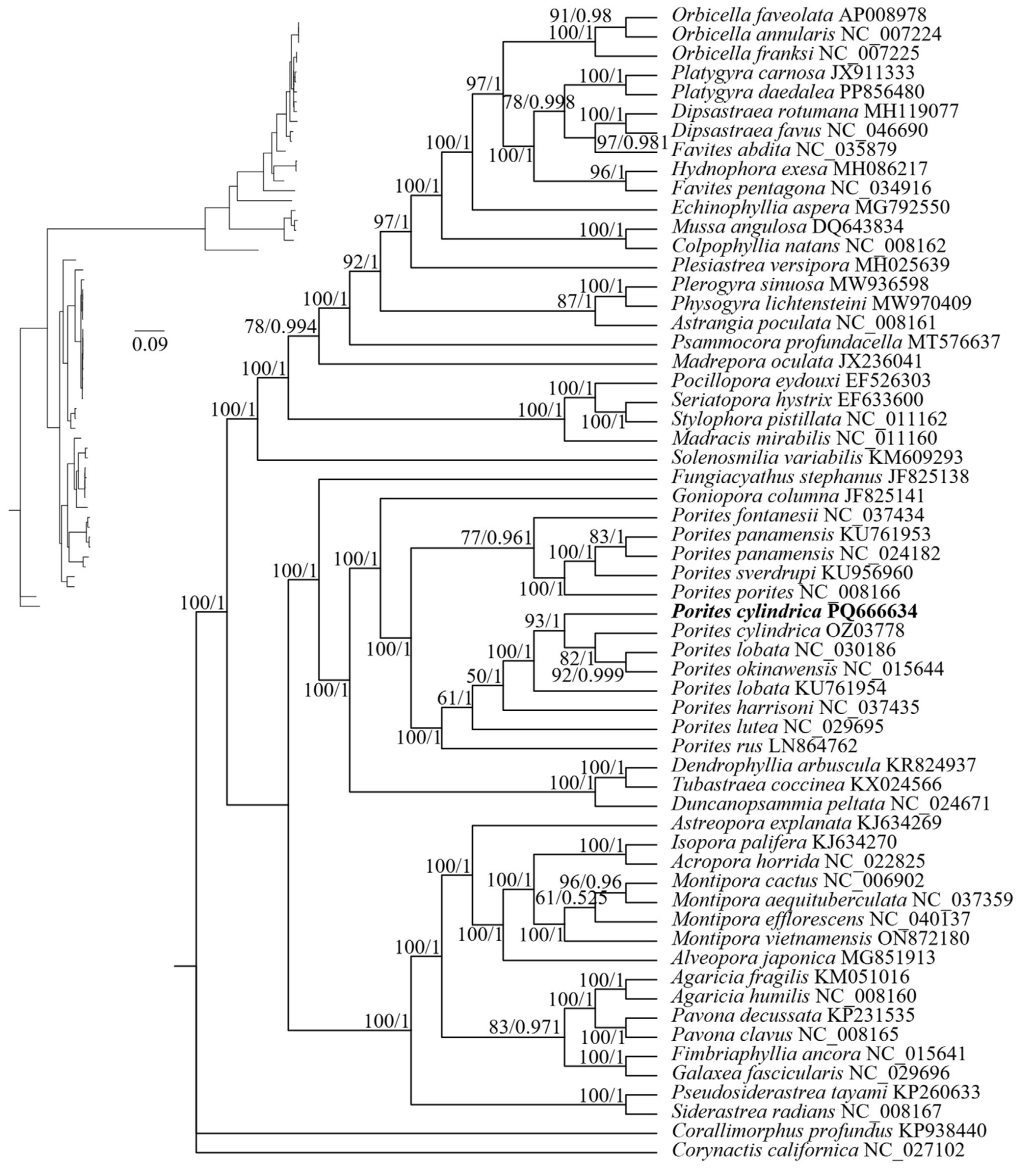
Phylogenetic tree based on 13 protein‐coding genes of 10 *Porites* species. Numbers on the right branches are posterior probability support values based on Bayesian inference, and numbers on the left branches are bootstrap support values from maximum parsimony analysis.

A phylogeny was reconstructed using ML and BI based on the concatenated sequences of 13 mitochondrial protein‐coding genes from 10 *Porites* species (13 mitogenomes) to determine the evolutionary position of *P. cylindrica* . The ML and BI analyses yielded highly congruent topologies (Figure 6), with strong nodal support throughout. All *Porites* species formed a maximally supported monophyletic group (BP = 100, PP = 1.0), within which two lineages were identified. The first lineage comprised *P. panamensis* , *P. sverdrupi* , *P. porites* , and *P. fontanesii*. The second lineage included *P. lobata* , *P. okinawensis* , *P. cylindrica* , *P. rus* , *P. lutea* , and *P. harrisoni* . Within this latter lineage, *P. cylindrica* was resolved as most closely related to *P. okinawensis* and *P. lobata* .

### 3.6 Nucleotide Diversity Analysis

**FIGURE 7:**
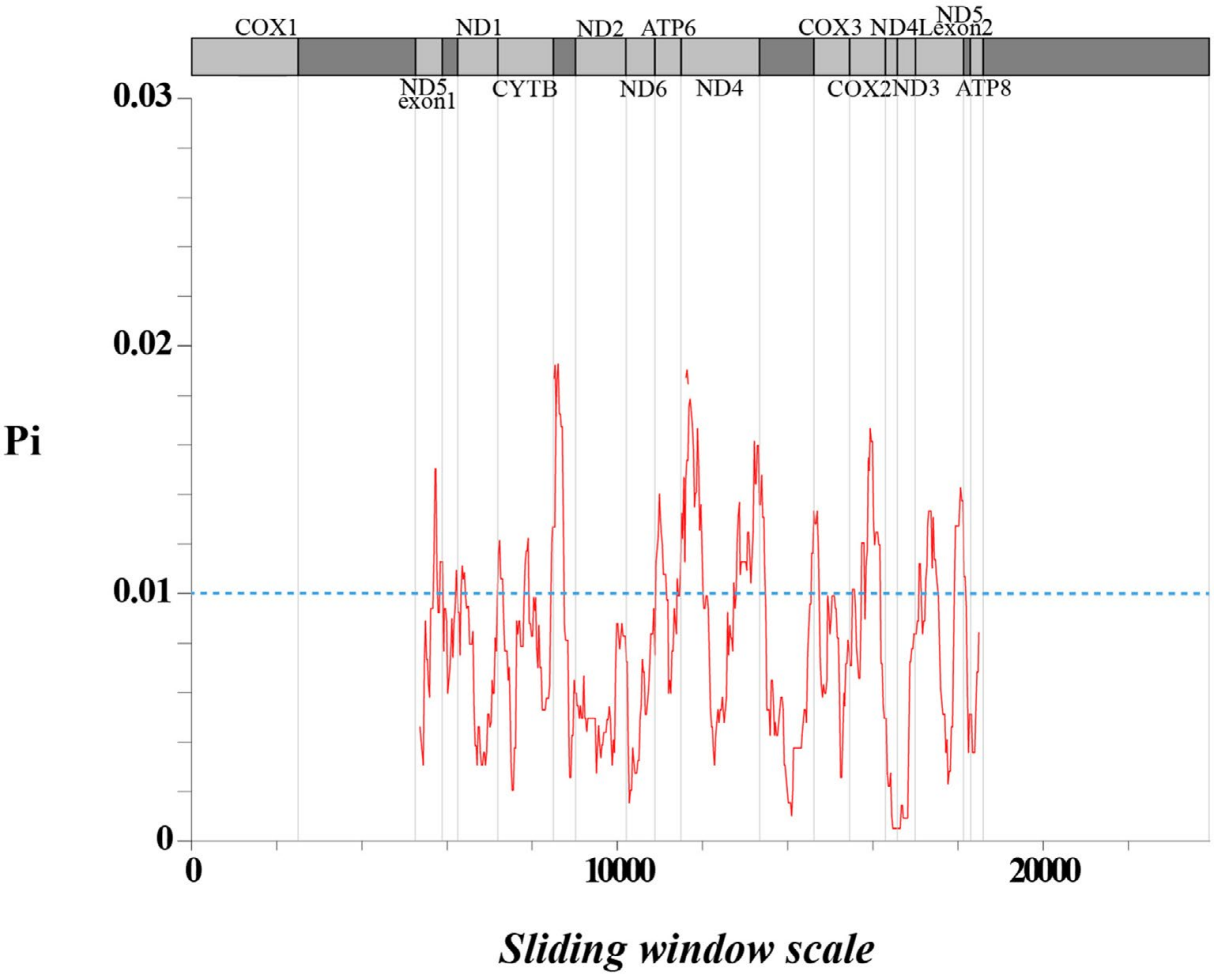
Nucleotide diversity analysis of 13 complete mitochondrial genomes of 10 *Porites* species. The blue dotted line marks the Pi values at 0.010.

Nucleotide diversity (Pi) indicates differences in nucleotide sequences between species and populations based on a selection of genomic regions with high variability as potential molecular markers for the population. The results of the Pi analysis showed that the mitochondrial fragment with the highest variability was CYTB‐trnM. Among the 10 species of *Porites*, 16 highly variable regions were identified (Pi value > 0.010): ND5‐exon1, trnE‐ND1, ND1, ND1‐CYTB, CYTB, CYTB‐trnM, trnM, ATP6, ATP6‐ND4, ND4, ND4‐rrnS, rrnS, rrnS‐COX3, COX3, COX2, and ND5‐exon2 (Figure 7; Table S4).

### 3.7 PCG Selection Pressure Analysis

To characterize the evolutionary pressure in PCGs of the 13 *Porites* mitochondrial genomes, Ka/Ks values of 13 PCGs were calculated (Table S5). The Ka/Ks values of all genes were less than 1, indicating that these genes had undergone purifying selection and suggesting that they exhibited relatively stable protein function. Among them, the Ka/Ks values of *COX1* in *P. okinawensis* and *P. lobata* were the highest at 0.49. The Ka/Ks values of *ND3* and *ND4L* were the lowest and almost undetectable.

## 4 Discussion

### 4.1 Mitogenome Assembly and Annotation of *P. cylindrica*

This study provides a newly sequenced, accurately assembled, and comprehensively annotated mitochondrial genome for *P. cylindrica* , which resolves ambiguities surrounding the structure of the only previously available sequence for this species. The sequence previously available in GenBank (accession OQ037789) was annotated as a linear molecule, which conflicts with the canonical circular architecture universally reported for scleractinian corals. Furthermore, it lacked detailed structural annotation. To address this, we performed de novo assembly of the mitogenome from the Xisha Islands specimen, which unequivocally confirmed its circular topology. We then provided a complete and validated structural annotation, precisely mapping all 13 protein‐coding genes, 2 rRNAs, and 2 tRNAs (GenBank accession PQ666634). This effort not only corrects the archival record but also provides a reliable, high‐quality reference genome. The confirmed circular architecture reinforces this conserved genomic feature in reef‐building corals and, crucially, establishes an essential and accurate foundation for all subsequent comparative mitogenomic analyses presented in this work.

### 4.2 Characteristics of *Porites* Mitochondrial Genomes

The mitochondrial genomes of the 10 *Porites* species exhibited high conservation in size (18,628–18,658 bp), GC content (36.20%–36.64%), and the canonical set of 13 PCGs and 2 rRNAs, consistent with reports from other reef‐building corals (Jia et al. 2025; Niu, Huang, et al. 2016; Niu, Lin, et al. 2016; Niu et al. 2020; Tian et al. 2022; Wang et al. 2013). The strong AT‐richness of *Porites* mitogenomes provides a potential contextual background for the observed codon bias toward A‐ and T‐ending codons in their PCGs. This bias might be partly understood in light of a mitogenomes‐wide AT‐rich environment, a pattern also common among other reef‐building corals (Dixon et al. 2016; Jia et al. 2025; Niu et al. 2020; Tian et al. 2021, 2022; Tian and Niu 2017; Tong et al. 2019; Shi et al. 2016). This explanation is further reinforced by the fact that most reef‐building corals possess only two tRNA genes in their mitochondrial genomes (Jia et al. 2025). In such a minimalist system, the classical model of codon usage that is optimized for tRNA abundance cannot be applied (Gissi et al. 2008; Lavrov and Pett 2016). Instead, the mutational bias itself becomes the predominant evolutionary force that systematically shapes codon choice by saturating the degenerate third positions with A and T nucleotides (Dixon et al. 2016; Tian et al. 2021). The primary role of the two tRNAs is likely to ensure basic translational functionality through extensive wobble pairing, rather than to drive selective codon preferences (Elahi and Prigge 2025; Yu and Li 2011).

Notably, we observed heterogeneity in tRNA gene copy number across the 13 mitogenomes, ranging from 1 to 12. This variation occurred independently of the species' geographic distribution range, as many widespread species (e.g., *P. lobata* ) retain only the minimal canonical pair. The evolutionary forces maintaining this diversity are not yet clear but may result from the combined effects of historical population size, mutation pressure, and the efficiency of mito‐nuclear co‐evolution.

### 4.3 Phylogenetic Relationships and Cryptic Diversity

The taxonomy of *Porites* has traditionally been based on morphological features; however, molecular evidence is also needed due to the genus' ultra‐high levels of phenotypic variation and morphological plasticity (Emblem et al. 2011; Forsman et al. 2017). Previous studies have investigated the phylogenetic relationship of *Porites* based on nuclear and mitochondrial gene fragments (Combosch et al. 2024; Prada et al. 2014); however, information on *P. cylindrica* samples from the South China Sea is lacking. In our study, ML and BI phylogenetic trees were constructed based on 57 reef‐building coral species, and the phylogenetic position of *P. cylindrica* was determined using molecular sequences. *Porites* are monophyletic, with species within the genus divided into two clades and having an extremely high support rate (BS = 100%, PP = 1), with *P. cylindrica* clustered with *P. lobata* NC_030186 and *P. okinawensis* . However, we observed that *P. cylindrica* PQ666634 and *P. cylindrica* OZ037789 formed two clades, indicating genetic divergence between *P. cylindrica* distributed in different regions. In the gene rearrangement analysis, the gene arrangement patterns of samples belonging to the same species differed, for example, in *P. lobata* . In the collinearity analysis, samples belonging to the same species did not cluster together. The possible causes of this may be the difficulty of species classification based on *Porites* morphology or significant genetic differentiation in samples collected from different regions (Fukami et al. 2004; Ladner and Palumbi 2012). This implies that current morphology‐based taxonomy may underestimate true biodiversity within *Porites*. Such cryptic lineages could represent distinct evolutionary significant units with unique adaptations to their local environments. Consequently, conservation strategies that treat morphospecies as homogeneous units may fail to protect this hidden genetic diversity, which could be crucial for the genus's resilience to environmental change.

### 4.4 Comparative of Variable Mitochondrial Markers in *Porites*

Identifying effective molecular markers for species delimitation in the coral genus *Porites* remains a significant challenge due to the slow evolutionary rate of its mitochondrial DNA. DNA barcodes, such as the cytochrome c oxidase I (COI) gene, can serve as standard short gene region markers for rapid, accurate, and efficient identification of many animal species (Dawnay et al. 2007; Che et al. 2012; Porter and Hajibabaei 2018). However, in scleractinian corals, the slow evolutionary rate of mitochondrial DNA often renders the COI gene insufficient for resolving closely related species (Huang et al. 2008; Wares 2014). This limitation necessitates the exploration of alternative, more variable mitochondrial regions. In this study, the results revealed 16 such highly variable regions (Pi > 0.010) that can serve as effective molecular markers for *Porites*. Several of these 16 highly variable mitochondrial regions were also reported as diversity loci by Combosch et al. (2024) and Paz‐García et al. (2016). First, the high variability observed in ND5 (exon1, exon2), ND4 (including ND4‐rrnS), rrnS (including rrnS‐COX3), and COX3‐COX2 in this study directly corresponds to the four core barcoding markers (MT20: ND5‐ATP8; MT12: ND4‐12S rRNA; MT16: COX3‐COX2; MT09: ND6‐ATP6) selected by Combosch et al. (2024). Second, the highly variable regions of ND1 and CYTB identified here align with the “core highly variable regions of the *Porites* mitogenome (16S rRNA, ND5, ND4, COX1, ND1, CYTB)” indicated by Paz‐García et al. (2016). Their team emphasized that the level of variation in these regions is 3–10 times higher than that in other reef‐building corals such as *Acropora* and *Pocillopora*, underscoring the universal value of these regions for species identification and phylogenetic studies in *Porites* (Paz‐García et al. 2016). Collective evidence from independent studies converges to highlight that these regions of the mitochondrial genome represent promising molecular tools for species delimitation and population genetic analyses within this taxonomically challenging genus.

## 5 Conclusions

This study presents a newly assembled and annotated mitochondrial genome for *P. cylindrica* from the Xisha Islands (PQ666634). It corrects the erroneous linear annotation in the previous record and confirms the canonical circular architecture conserved across scleractinian corals.

Comparative analysis of mitogenomes from 10 *Porites* species revealed profound conservation in size, structure, and core gene content, coupled with strong AT‐biased mutation pressure shaping codon usage. Furthermore, our phylogenetic analyses uncovered significant genetic divergence among geographically separated populations morphologically identified as the same species (e.g., *P. cylindrica* , *P. lobata* ). These findings strongly suggest the presence of extensive cryptic diversity within the genus, indicating that morphology‐based taxonomy likely underestimates true species boundaries and evolutionary units in *Porites*.

Finally, we identified and cross‐validated 16 highly variable mitochondrial regions as promising molecular markers for species delimitation in this taxonomically challenging genus. The convergence of our results with those of independent studies underscores the robustness of these markers for future phylogenetic and population genetic research. Collectively, this work provides an essential mitogenomic resource reveals patterns of cryptic diversity with implications for conservation, and offers practical tools for refining the systematics of *Porites*.

## Author Contributions

**Shuwen Jia:** conceptualization (equal), data curation (equal), formal analysis (equal), funding acquisition (equal), investigation (equal), methodology (equal), project administration (equal), resources (equal), software (equal), supervision (equal), validation (equal), visualization (equal), writing – original draft (equal), writing – review and editing (equal). **Tongtong Shen:** data curation (equal), formal analysis (equal), writing – original draft (equal). **Wenqi Cai:** investigation (equal), resources (equal). **Jian Zhang:** investigation (equal), resources (equal). **Yi Wang:** data curation (equal), formal analysis (equal). **Zefu Cai:** data curation (equal), formal analysis (equal). **Jie Shen:** data curation (equal), formal analysis (equal). **Shiquan Chen:** conceptualization (equal), data curation (equal), formal analysis (equal), funding acquisition (equal), investigation (equal), methodology (equal), project administration (equal), resources (equal), software (equal), supervision (equal), validation (equal), visualization (equal), writing – original draft (equal), writing – review and editing (equal).

## Funding

This work was supported by the National Natural Science Foundation of China (42166006), Department budget projects of Hainan province in 2024, and Hainan provincial Natural Science Foundation of China (424RC538, ZDYF2024SHFZ146, 321MS0810, 421RC1106).

## Conflicts of Interest

The authors declare no conflicts of interest.

## Supporting information


**Figure S1:** Sequencing coverage plot of the mitochondrial genome.

**Figure S2:** Gene annotation of the published linear *Porites cylindrica* mitogenome (OZ037789) for comparative analysis.

**Table S1:** Microsatellite sequence information for 13 complete mitochondrial genomes of 10 *Porites* species.

**Table S2:** Long repeat sequence information for 13 complete mitochondrial genomes of 10 *Porites* species.

**Table S3:** Synonymous codon usage based on 13 PCGs of 13 complete mitochondrial genomes of 10 *Porites* species.

**Table S4:** Nucleotide diversity (Pi) values for 13 complete mitochondrial genomes of 10 *Porites* species.

**Table S5:** Ka/Ks values based on 13 PCGs of 13 complete mitochondrial genomes of 10 *Porites* species.

## Data Availability

The data generated in this study have been deposited as follows: the mitochondrial genome in GenBank under accession PQ666634, and the raw sequencing data in the SRA under BioProject PRJNA1191632 and accession SRX26937562. The custom Python script used for analysis is available in a GitHub repository: https://github.com/jiashuwen/Draw_SequencingDepth.
