# Supplementary material for: Complete Mitochondrial Genome of Porites cylindrica From the Xisha Islands: Characterization and Comparative Mitogenomics of the Genus
Source: Ecol Evol. 2026 Mar 18;16(3):e73297. doi: 10.1002/ece3.73297 (PMC13093563; doi:10.1002/ece3.73297)
Supplement: Supplementary file 1 — Figure S1: Sequencing coverage plot of the mitochondrial genome. [file ECE3-16-e73297-s001.docx]

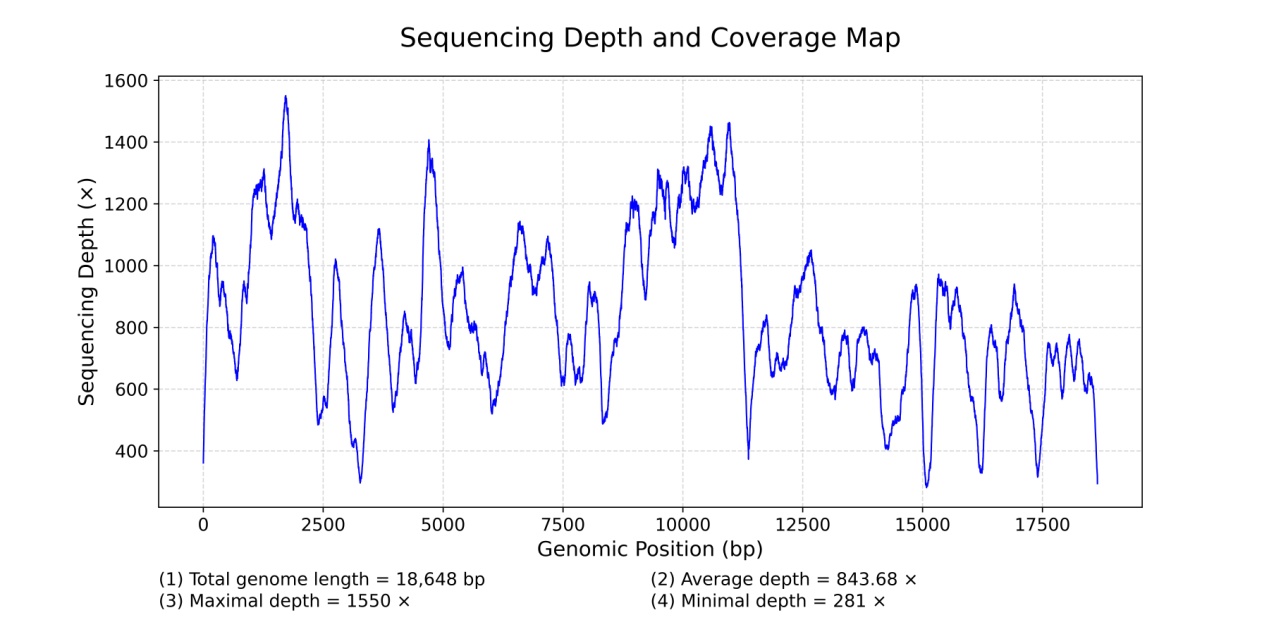


Fig. S1. Sequencing Coverage Plot of the mitochondrial genome. This plot was generated using a custom Python script. The x-axis represents the positions of the mitochondrial genome, and the y-axis represents the corresponding sequencing depth. Relevant results have been added to the Results section as suggested.
