# Supplementary figures and images for: Complete Mitochondrial Genome of Porites cylindrica From the Xisha Islands: Characterization and Comparative Mitogenomics of the Genus
Source: Ecol Evol. 2026 Mar 18;16(3):e73297. doi: 10.1002/ece3.73297 (PMC13093563; doi:10.1002/ece3.73297)

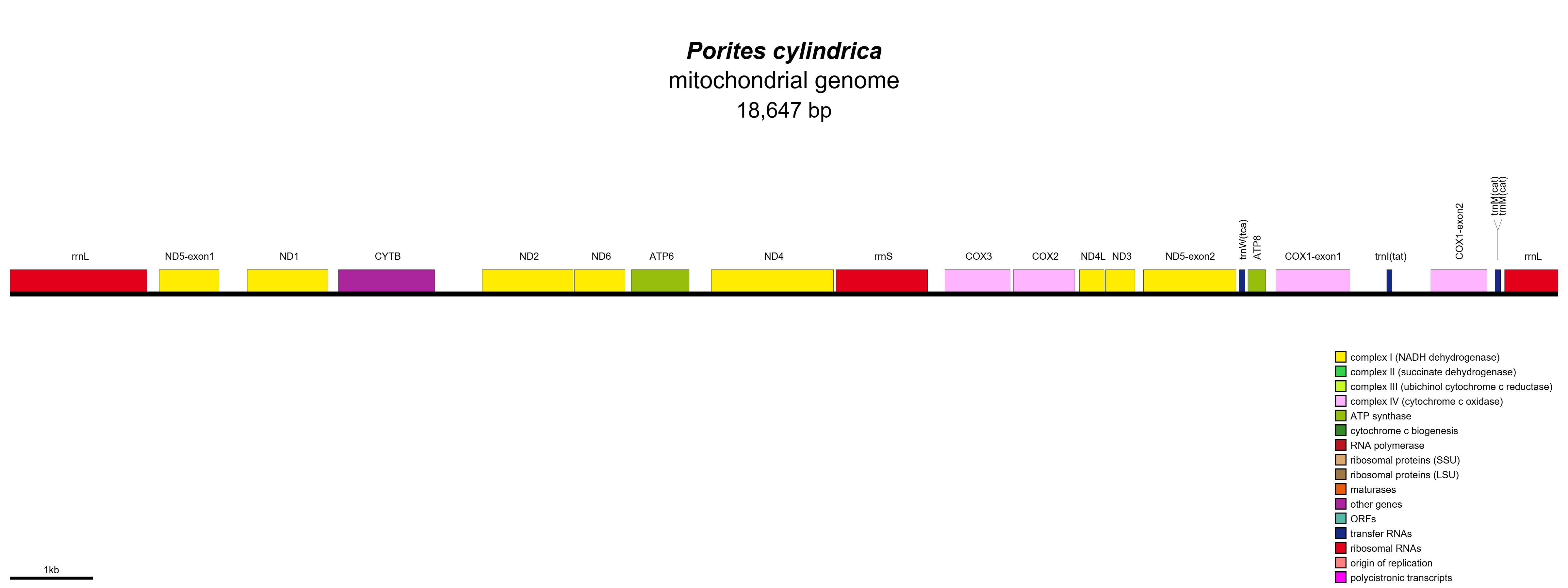

Supplement: Supplementary file 2 — Figure S2: Gene annotation of the published linear Porites cylindrica mitogenome (OZ037789) for comparative analysis. [file ECE3-16-e73297-s002.jpg]
